# Definition of bulky disease in early stage diffuse large B-cell lymphoma in computed tomography on coronal and transverse planes

**DOI:** 10.3389/fonc.2023.1063438

**Published:** 2023-11-28

**Authors:** Mohammad Ma’koseh, Heba Farfoura, Yumna Khatib, Zaid Omari, Hazim Ababneh, Baha A. Fayoumi, Ayat Taqash, Mohammad Al-Rwashdeh, Alaa Abufara, Omar Shahin, Khalid Halahleh, Kamal Al-Rabi

**Affiliations:** ^1^ Department of Internal Medicine, King Hussein Cancer Center, Amman, Jordan; ^2^ School of Medicine, The University of Jordan, Amman, Jordan; ^3^ Department of Radiology, King Hussein Cancer Center, Amman, Jordan

**Keywords:** ESDLBL, CT coronal plane, CT transverse plane, bulky disease, combined modality treatment

## Abstract

**Background:**

In early stage diffuse large B-cell lymphoma (ESDLBL), tumor bulkiness is an important determinant of treatment and prognosis. Tumor bulk is usually measured on transverse computed tomography (CT) plane and variably defined from 5 to 10 cm.

**Objectives:**

Our study aims to investigate the prognostic significance of bulky disease measured on CT coronal and transverse planes and to evaluate the outcome of patients with bulky disease.

**Methods:**

Patients with ESDLBL and treated with rituximab, cyclophosphamide, doxorubicin, and prednisolone (RCHOP) with or without radiotherapy were included. Receiver Operating Characteristic (ROC) analysis was used to identify the optimal tumor dimension that correlated with progression, relapse, or death. Correlation between different variables and progression-free survival (PFS) and overall survival (OS) were analyzed using log-rank (Mantel–Cox) test and Cox proportional hazard models.

**Results:**

A total of 127 patients with a median age of 47 (range: 18–90) years were included. Eighty-two (64.6%) patients treated with combined modality treatment (CMT) [RCHOP + radiotherapy]. After a median follow-up of 40 (range: 2–114) months, 3-year PFS and OS were 83.9% (95% CI: 76.759%–89.981%), and 80.6% (95% CI: 72.499%–87.531%), respectively. Tumor dimension of >7.5 cm measured on either CT plane was the optimal cutoff point to define bulky disease. Three-year PFS and OS were inferior in the group of patients with no bulky disease on transvers plane (*n* = 84) but had bulky disease on coronal plane (*n* = 9,10.7%); (94.2% vs. 75%, *p* = 0.017 and 90.5% vs. 56.3%, *p* = 0.002), as well as in patients with no bulky disease on coronal plane (*n* = 89), but had bulky disease on transverse plane (*n* = 14, 15.7%); (94.1% vs. 62.3%, *p* < 0.001, and 90.4% vs. 63.5%, *p* = 0.002). Compared to RCHOP alone, 3-year PFS and OS were better in patients with bulky disease treated with CMT (78% vs. 52.5%, *p* = 0.018 and 81.8% vs. 38.7%, *p* = 0.003) but not in patients with non-bulky disease (96.2% vs. 93%, *p* = 0.691 and 87.6% vs. 91.5%, *p* = 0.477).

**Conclusion:**

In ESDLBL, measurement of tumor mass on transverse and coronal CT planes may help in better identification of patients with bulky disease. The use of CMT was associated with better survival outcomes in patients with bulky disease.

## Introduction

Diffuse large B-cell lymphoma (DLBL) is the most common subtype of non-Hodgkin lymphoma (NHL) representing about 30%–40% of all lymphomas. About 40% of DLBL present as early stage disease, defined as one or two lymph node areas above or below the diaphragm that can be contained in one radiation field (1, 2).

In early stage DLBL (ESDLBL), tumor bulkiness and stage-adjusted international prognostic index (sa-IPI) are the main determinants of prognosis and treatment (3, 4). Patients with non-bulky ESDLBL are usually treated with abbreviated course of chemotherapy (three to four cycles) followed by radiotherapy or six cycles of chemotherapy, while those with bulky disease are usually treated with six cycles of chemotherapy followed by radiotherapy (5, 6). Recently, the LYSA/GOELAMS, S1001 and FLYER trials established that in patients with non-bulky ESDLBL, achievement of complete metabolic response after 3–4 cycles of chemotherapy, obviated the need for consolidative radiotherapy (7, 8) or completing six cycles of chemotherapy (9).

However, definition of bulky disease remains variable. Tumor diameters from 5 to 10 cm were used in different clinical trials. For example, in the MabThera International group study, increasing maximum tumor diameter above 5 cm was associated with linear prognostic effect, and a cutoff point of ten centimeters was finally selected (10). However, most of the patients included had an advanced stage disease. Also, in the S1001, Southwest oncology group (SWOG) 014 and in the Groupe d’Etudes des Lymphomes de l’Adulte 93-4 trials, bulky disease was defined as tumor mass measuring more than 10 cm in maximal diameter (8, 11, 12), while in another study conducted at MD Anderson Cancer Center, a cutoff point of 5 cm was used to define bulky disease (13). Finally, in the RICOVER-60 trial, 7.5 cm or more was used to define bulky disease (14).

Although the computed tomography (CT) plane used to measure tumor dimension to define bulky disease was not specified in clinical trials, radiologist often report it on transverse plane (15). In a study that evaluated the definition of bulky disease on CT planes in Hodgkin lymphoma, different cutoff points were found in coronal and transverse measurements (16).

Our study aims to evaluate the correlation between maximum tumor dimensions measured on CT scan transverse and coronal planes with outcomes and to evaluate the outcome of patients with bulky disease.

## Patients and methods

Patients with pathological diagnosis of DLBL; stage I or stage II disease; and treated with rituximab, cyclophosphamide, doxorubicin, and prednisolone (RCHOP) at our center from 2012 until 2019 were identified and data were extracted retrospectively using our hospital database and electronic medical records. Different demographic, laboratory, disease related, and treatment related variables were collected. Staging was done according to Lugano classification (2). Patients were staged with CT scan from 2012 to 2016 (*n* = 52) and with CT and positron emission tomography–CT (PET-CT) scan after 2016 (*n* = 85).

CT scans were performed on Philips ICT helical scanner. Images were reconstructed at 3 mm intervals in the Picture Archiving and Communication System. Using calipers with measurements in centimeters, two independent board-certified radiologists reviewed pre-treatment CT scans and measured maximal tumor diameter on transverse and coronal planes in any dimension. Patients with no measurable disease on both planes were excluded.

Sa-IPI was calculated based on age, serum lactate dehydrogenase, Eastern Cooperative Oncology Group (ECOG) performance status and stage (I vs. II or IIE), as previously described (4). Progression-free survival (PFS) and overall survival (OS) were defined as time from initiation of treatment till progression of disease, relapse, or death, respectively.

Response to initial treatment was determined using Lugano criteria (2).

We used the Receiver Operating Characteristic (ROC) analysis to identify the largest dimension measured on either CT plane that best correlates with progression or death. Then, we compared the PFS and OS for patients with tumor dimension below the cutoff point on one CT plane but had a dimension more than the cutoff point on the other CT plane.

Survival outcomes were plotted using Kaplan–Meier method. The associations between different variables with PFS and OS was analyzed using log-rank (Mantel–Cox) test in the univariate analysis and Cox proportional hazard models in the multivariate analysis.

## Results

### Patients and disease bulk

A total of 127 patients were included with a median age of 47 (range: 18–90) years, and a slight male predominance (58%). Fifty-one (40.2%) patients were having extra-nodal involvement. sa-IPI was 0–1 in 73 (57.4%). Eighty-two (64.6%) patients treated RCHOP followed by radiotherapy as combined modality treatment (CMT). One hundred four (81.9%) patients were given six to eight cycles of chemotherapy. Patients’ characteristics are detailed in **Table 1**.

**Table 1:** Patient’s characteristics.

Variable	Number (%)
Age < 60 years	92 (72.4%)
Age ≥ 60 years	35 (27.6%)
ECOG performance status	
0–1	116 (91.3%)
2 or more	11 (8.7%)
High LDH	49 (38.6%)
Stage	
I	49 (38.6%)
II	78 (68.4%)
Stage adjusted IPI	
0	23 (18.1%)
1	51 (40.2%)
2	37 (29.1%)
3	15 (11.8%)
4	1 (0.8%)
Extranodal localization	51 (40.2%)
Nodal localization	
Head and neck	33 (25.9%)
Mediastinal	24 (18.9%)
Axillary	4 (3.2%)
Abdominal/inguinal	15 (11.8%)
Number of chemotherapy cycles	
< 6	23 (18.1%)
6–8	104 (81.9%)
Radiotherapy	82 (64.6%)
Treatment	
3–4 cycles of chemotherapy with radiotherapy	21 (16.6%)
6 or cycles of chemotherapy with radiotherapy	61 (48%)
Chemotherapy only	45 (35.4%)
Maximum diameter on either CT plane	
≤ 7.5 cm	75 (59.1%)
>7.5 cm	52 (40.9%)
Maximum diameter on transverse plane	
≤ 7.5 cm	84 (66.1%)
>7.5 cm	43 (33.9%)
Maximum diameter on coronal plane	
≤ 7.5 cm	89 (70%)
>7.5 cm	38 (30%)

ECOG, Eastern Cooperative Oncology Group; LDH, lactate dehydrogenase; IPI, international prognostic index.

**Figure 1:**
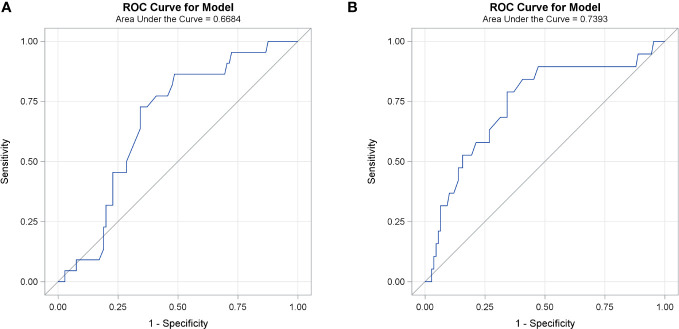
ROC curve for different cutoff points of maximal tumor dimension measured on both CT planes and death **(A)** and progression **(B)**.

The median tumor diameter on transverse plane was 6.3 (range: 1.1–16.4) cm and on coronal plane was 6.4 (range: 0.9–22) cm. The ROC curve identified that maximal tumor dimension measured on either CT plan of 7.6 cm is the optimal cutoff point that best correlates with progression (AUC 0.74, sensitivity 78.9% and specificity 65.7%) and death (AUC 0.67, sensitivity 73% and specificity 66.7%), **Figure 1**, and is used to define bulky disease. Accordingly, 43 (33.9%) patients were defined to have bulky disease on either CT plane.

Among the group with no bulky disease on the transverse plane (*n* = 84), nine (10.7%) had a bulky disease on the coronal plane. On the other hand, in patients with no bulky disease on coronal plane (*n* = 89), 14 (15.7%) had a bulky disease on the transverse plane.

### Outcomes according to disease bulk

**Figure 2:**
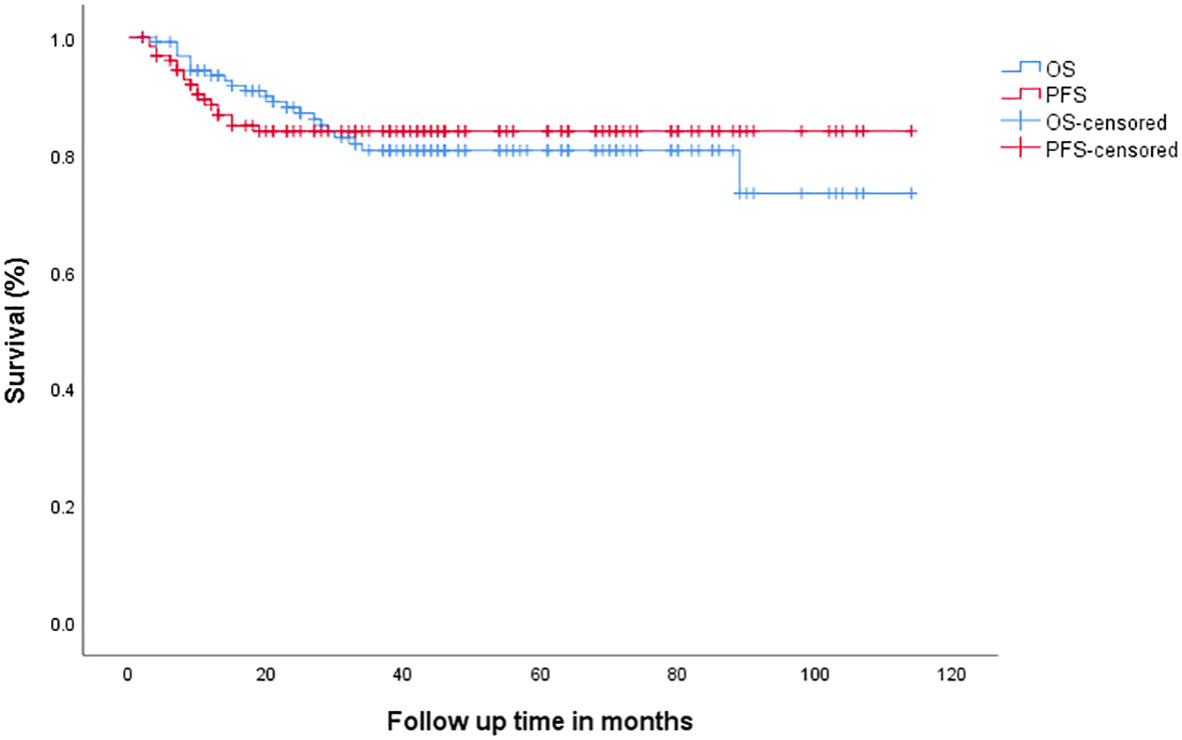
Overall survival (OS) and progression-free survival (PFS) in all patients.

After a median follow-up of 40 (range 2–114) months, 3-year PFS for all patients was 83.9% (95% CI: 76.759%–89.981%), and 3-year OS was 80.6% (95% CI: 72.499%–87.531%), **Figure 2**.

**Figure 3:**
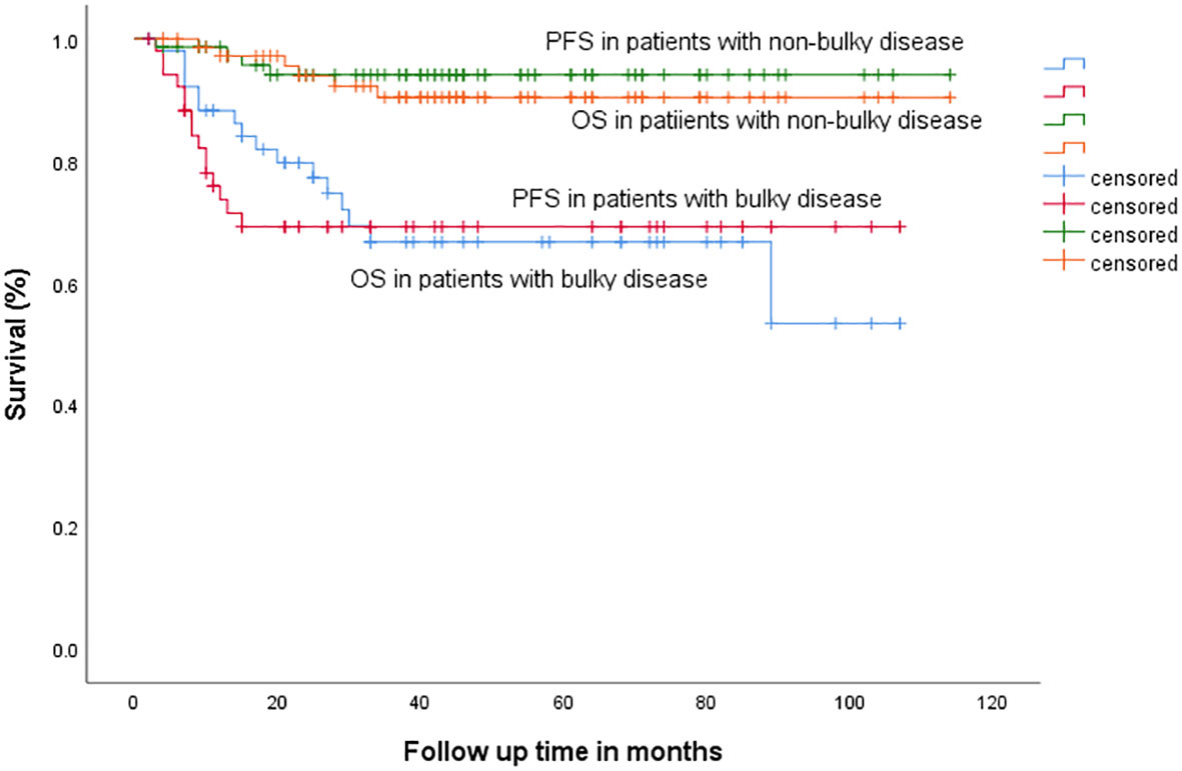
Progression-free survival (PFS) and overall survival (OS) in patients with bulky versus non-bulky disease.

Univariate analysis showed that bulky disease (*p* < 0.001) and sa-IPI >1 (*p* = 0.008) correlated with PFS. On the other hand, bulky disease (*p* = 0.001), sa-IPI >1 (*p* < 0.001) and radiotherapy (*p* = 0.017) correlated with OS, **Table 2** and **Figure 3**.

**Table 2:** Univariate analysis.

Variable	3-year PFS%	*P*-value	3-year OS %	*P*-value
Stage adjusted -IPI				
0–1 (*n* = 73) >1 (*n* = 53)	90.9% 74.1%	0.008	90.8% 67.1%	< 0.001
Tumor dimension ≤ 7.5 cm on either CT plane Tumor dimension > 7.5 cm on either CT plane	94.1% 69.2%	< 0.001	90.4% 66.7%	0.001
Extranodal				
Yes No	87.0% 81.8%	0.415	80.4% 80.7%	0.71
Radiotherapy				
Yes No	86.6% 78.8%	0.13	87.2% 68.1%	0.017
6 or more cycles of RCHOP Less than 6 cycles of RCHOP	84.5% 81.4%	0.751	83.9% 70%	0.185
Treatment				
3–4 cycles of chemotherapy with radiotherapy 6 or more cycles of chemotherapy with radiotherapy Chemotherapy only	84.9% 87.5% 78.8%	0.307	77% 91.2% 68.1%	0.024
3–4 cycles of chemotherapy with radiotherapy 6 or more cycles of chemotherapy with radiotherapy	87.5% 84.9%	0.765	77% 91.2%	0.109
Head and neck nodal disease (*n* = 33) Other nodal (*n* = 43)	87.2% 77.5%	0.234	89.8% 73.7%	0.116
Head and neck nodal disease (*n* = 33) No head and neck involvement (*n* = 94)	87.2% 82.9%	0.507	89.8% 81.9%	0.190
Mediastinal or axillary nodal disease (*n* = 28) Other nodal (*n* = 28)	78% 83.9%	0.459	50.9% 83.1%	0.402
Mediastinal or axillary nodal disease (*n* = 28) No mediastinal or axillary involvement (*n* = 99)	78% 85.6%	0.291	50.9% 84.6%	0.331
Abdominal or inguinal nodal (*n* = 15) Other nodal (*n* = 61)	75.5% 83%	0.521	68.6% 83.5%	0.373
Abdominal or inguinal nodal disease (*n* = 15) No abdominal or inguinal involvement (*n* = 112)	75.5% 84.9%	0.375	68.6% 88.3%	0.291

IPI, international prognostic index.

On multivariate analysis, bulky disease correlated with PFS (*p* = 0.004). OS correlated with bulky disease (*p* = 0.009), sa-IPI > 1 (*p* = 0.032) and radiotherapy (*p* = 0.019), **Table 3**.

**Table 3:** Multivariate analysis.

Progression-free survival				
Variable	*P*-value	HR	95% CI	
			Lower	Upper
Stage-adjusted-IPI Tumor dimension > 7.5 cm on either CT plane	0.08 0.004	3.062 8.080	0.899 1.664	6.534 15.984
Overall survival				
Stage-adjusted-IPI Tumor dimension > 7.5 cm on either CT plane Radiotherapy	0.032 0.009 0.019	3.14 3.699 2.862	1.016 1.389 1.189	8.916 9.856 6.887

IPI, international prognostic index.

**Figure 4:**
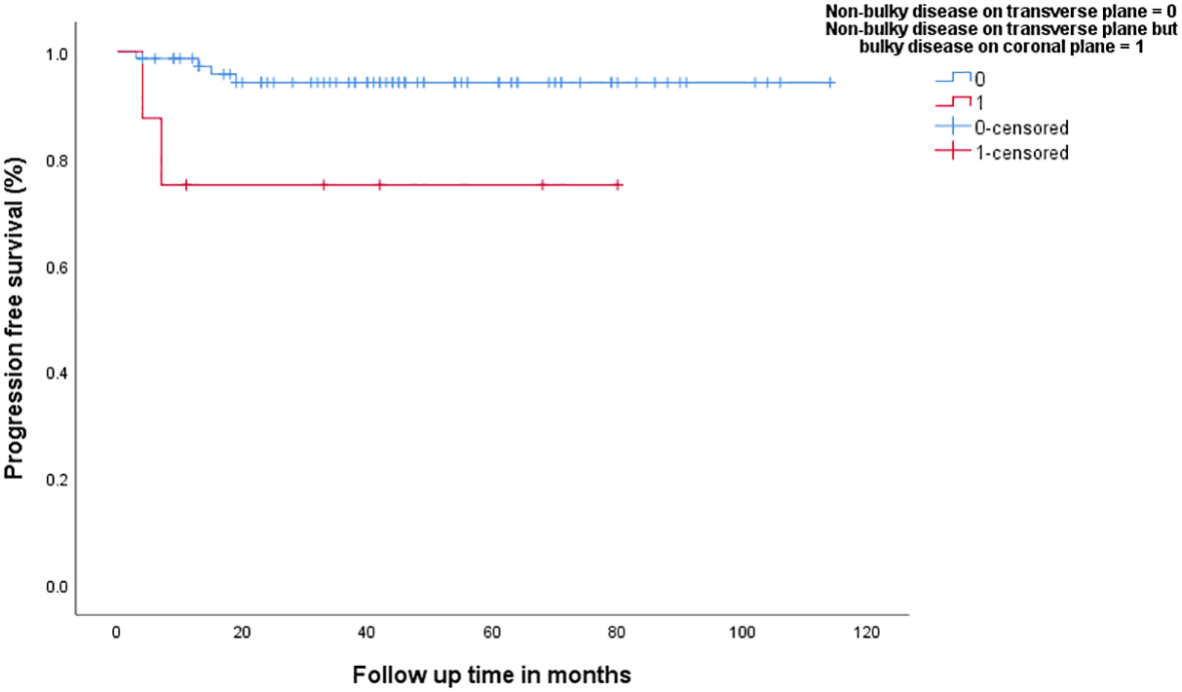
Progression-free survival in patients with non-bulky disease on transverse plane versus patients with bulky-disease on coronal plane but no-bulky disease on transvers plane.

**Figure 5:**
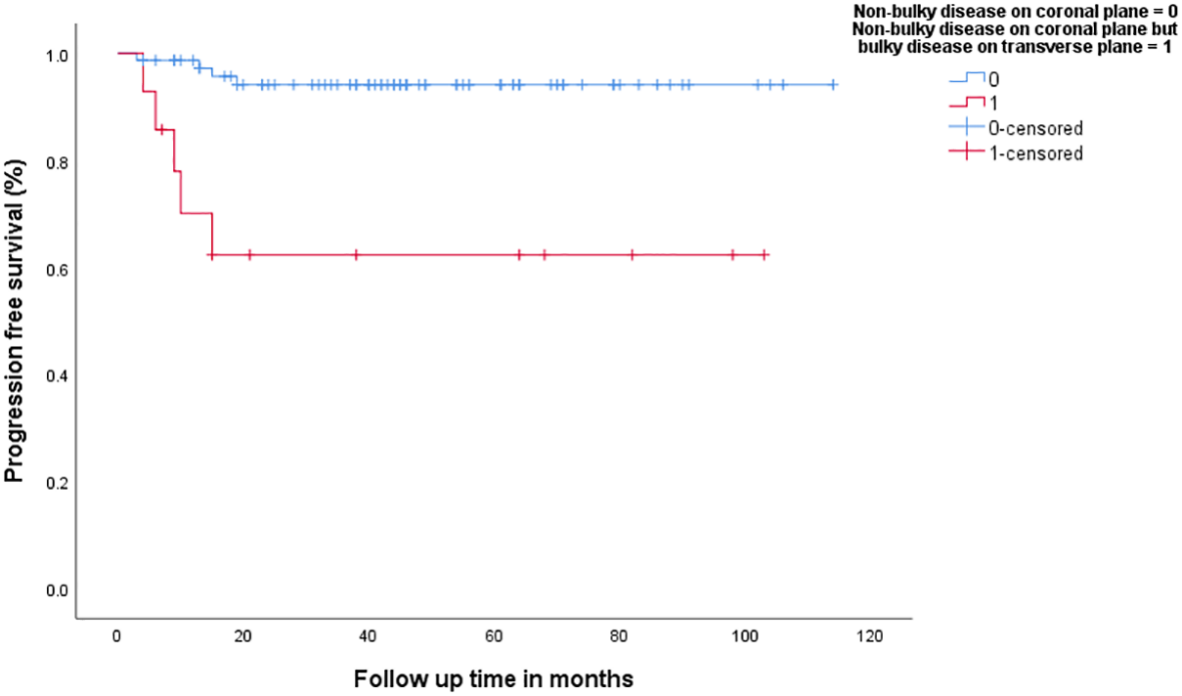
Progression-free survival in patients with non-bulky disease on coronal plane versus patients with bulky-disease on transvers plane but no-bulky disease on coronal plane.

In patients with no bulky disease on transverse plane (*n* = 84), the group that had bulky disease on longitudinal plane (*n* = 9, 10.7%) had inferior 3-year PFS (94.2% vs. 75%, *p* = 0.017), **Figure 4**, as well as 3-year OS (90.5% vs. 56.3%, *p* = 0.002). In addition, patients with no bulky disease on coronal plane (*n* = 89), the group that had bulky disease on transverse plane (*n* = 14), had inferior 3-year PFS (94.1% vs. 62.3%, *p* < 0.001), **Figure 5**, as well as 3-year OS (90.4% vs. 63.5%, *p* = 0.002).

### Outcomes according to treatment modality

**Figure 6:**
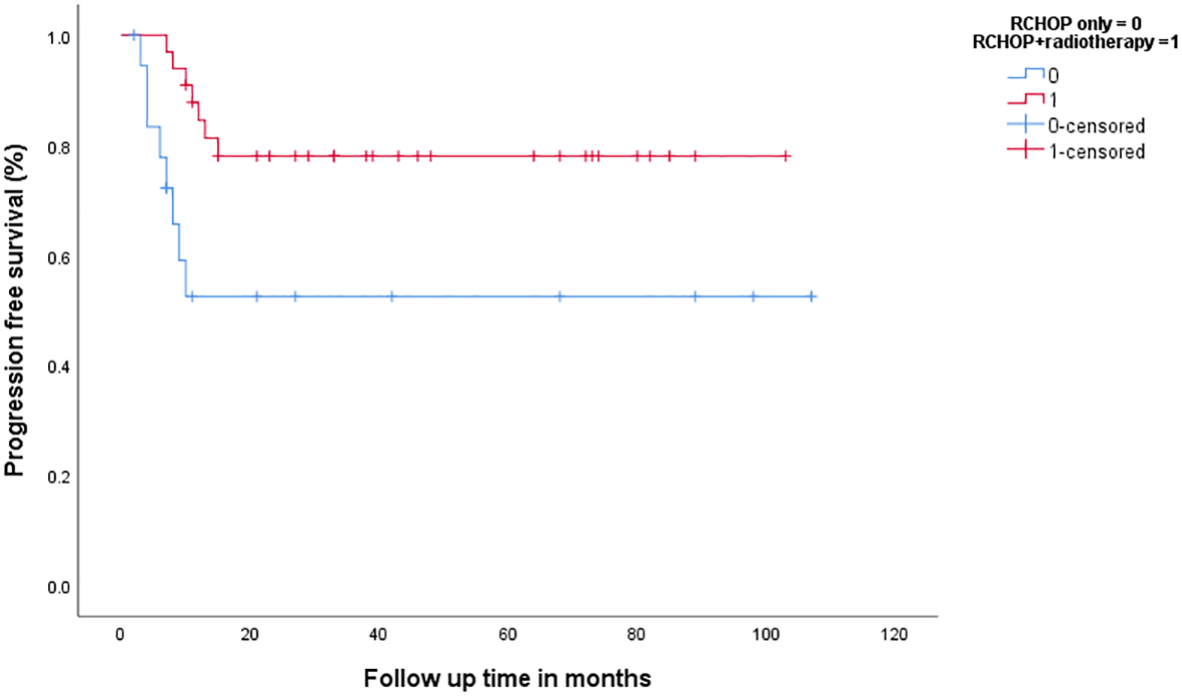
Progression-free survival in patients with bulky disease according to the treatment modality.

**Figure 7:**
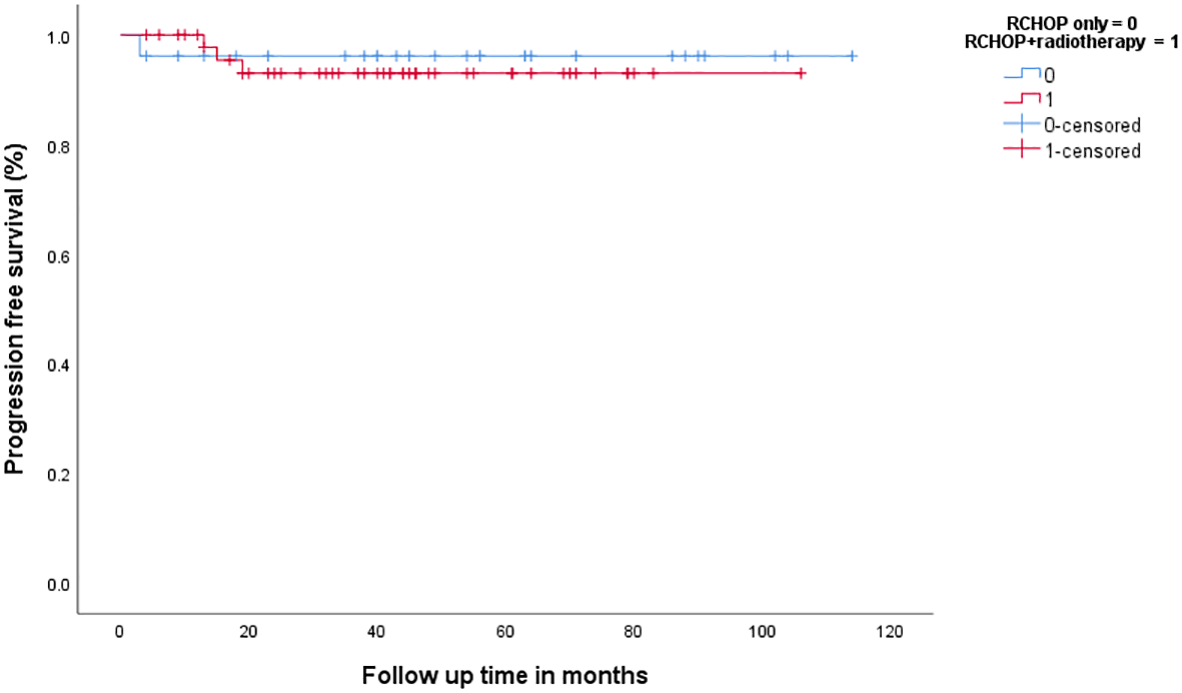
Progression-free survival in patients with non-bulky disease according to the treatment modality.

In patients with bulky disease on either CT plane (*n* = 52), 33 (63.5%) were treated with CMT and 19 (36.5%) were treated with RCHOP alone (three were given four cycles of RCHOP and 16 were given six to eight cycles). Administration of CMT, compared to chemotherapy alone, was associated with a better 3-year PFS (78% vs. 52.5%, *p* = 0.018), **Figure 6**, and 3-year OS (81.8% vs. 38.7%, *p* = 0.003). On the other hand, 75 patients with no bulky disease on either CT plane; 26 (34.6%) were treated with chemotherapy (25 were given six to eight cycles of RCHOP), and the rest were given CMT. There was no difference in 3-year PFS or OS between the chemotherapy and CMT (3-year PFS: 96.2% vs. 93%, *p* = 0.691, 3-year OS: 87.6% vs. 91.5%, *p* = 0.477, respectively), **Figure 7**.

In the group of patients treated with chemotherapy only (*n* = 45), 43 (95.5%) were given six to eight cycles of RCHOP. 19 (42.2%) had bulky disease. Three-year PFS and OS were significantly inferior in patients with bulky disease (3-year PFS: 96.2% vs. 56.2%, *p* = 0.001, 3-year OS: 87.6% vs. 38.7%, *p* = 0.001). Death resulted from causes not related to relapse in two patients with non-bulky disease and three patients with bulky disease.

On the other hand, in the group of patients treated with CMT (*n* = 82), 33 (37%) had bulky disease. Three-year PFS was inferior in patient with bulky disease (93% vs. 78%, *p* = 0.039). However, there was no difference in 3-year OS (91.5% vs. 81.8%, *p* = 0.149). Four patients (three with bulky disease and one with non-bulky disease) died with no relapse.

## Discussion

Refining the definition of bulky disease on multidimensional CT scan in ESDLB is important in the view of the evolving data on elimination of radiotherapy and optimizing the number of chemotherapy cycles. To our knowledge, this is the first study that independently assessed the value of measuring tumor bulky in transverse and coronal planes in ESDLB.

In the era of multidimensional CT scan, measurement of tumor mass can be done on transverse, coronal or sagittal planes. Because three-dimensional or volumetric measurements are difficult to assess, and is rarely reported, measurements are often reported on the transverse plane only. In our study, a tumor dimension of >7.5 cm on either CT plane is the optimal cutoff point that correlated with progression and death. This is consistent with the results of RICOVER trial (14) and close to the 7-cm cutoff point used to define bulky disease, which is used to exclude patients with bulky disease in the most recent trails that used PET-CT adapted approach to eliminate radiotherapy in LYSA/GOELAMS trial (7) or to optimize the number of chemotherapy cycles in the FLYER trial (8).

In our study, about 10%–15% of patients with no bulky disease on either CT plane were found to have bulky disease on the other plane. This group had worse PFS and OS. This finding highlights the importance of measuring tumor dimension on either CT plane when deciding on the treatment approach in patients with ESDLBL.

Consistent with the results of LYSA/GOELAMS, S1001 and FLYER trials (7–9), our study showed that the use of CMT in patients with non-bulky ESDLBL was not associated with better survival outcomes and confirmed the excellent PFS outcomes with treatment of chemotherapy in this group (3-year PFS: 96.2%). Although PET-CT adapted approach was not used in our patients, our results support the current approach of omitting radiotherapy in this context.

The outcomes of our patients with bulky disease compares favorably to the results of a recent study from Finland (3-year PFS of 69.2% vs. 2-year PFS of 53%), possibly because of the relatively younger age of our patients (47 vs. 68 years), and the fact that higher percentage received radiotherapy (63.3% vs. 44.3%) (17).

In our study, bulky disease was the main predictor of worse PFS and OS in patients treated with chemotherapy only, independent of sa-IPI. Although patients with bulky disease treated with CMT, compared to chemotherapy only, had a better PFS and OS, the negative effect of bulky disease on PFS was still persistent in the group who received CMT, possibly indicating that addition of radiotherapy to this group may not be enough to optimize their outcomes. More studies would be necessary to identify the best treatment approach is these patients.

The positive impact of radiotherapy on survival outcomes in patients with ESDLBL was also reported in other studies (12, 13, 18, 19) but was not supported by the results of the UNFOLDER trial in patients with low age-adjusted IPI and bulky disease of more than 7.5 cm (20) and in a recent metanalysis (21). The role radiotherapy in the management of ESDLBL is being better defined in the era of PET-CT adapted approach.

Another special population to consider in ESDLBL is extranodal disease. Compared to patients with nodal disease, studies reported conflicting outcomes (22). Our study showed no difference in PFS or OS between nodal and extranodal disease, similar to the results of a recent SWOG analysis (23). On the other hand, another retrospective study reported that, in patients with stage I disease, extranodal disease had inferior outcomes (24).

The location of lymphadenopathy was reported to correlate with outcomes in ESDLBL. Although head and neck location had the best outcomes, abdominal or pelvic had the worst (25). Our study showed similar results, but the difference was not statistically significant, possibly because of the small sample size.

We admit that our study has important limitations including the retrospective design and the lack of data on cell of origin and double expressor and double hit classifications, because these tests were not routinely done in our center during the study period.

## Conclusion

Bulky disease has a significant impact on survival outcomes in ESDLBL. Measurement of tumor dimensions on transverse and coronal CT planes may help in better identification of bulky disease and optimizing treatment approaches. In patients with bulky ESDLBL, the use of CMT, compared to chemotherapy only, was associated with better outcomes.

## Data availability statement

The raw data supporting the conclusions of this article will be made available by the authors, without undue reservation.

## Ethics statement

The studies involving humans were approved by Institution review board -King Hussein Cancer Center - Amman-Jordan. The studies were conducted in accordance with the local legislation and institutional requirements. Written informed consent for participation was not required from the participants or the participants’ legal guardians/next of kin in accordance with the national legislation and institutional requirements.

## Author contributions

All authors listed have made a substantial, direct, and intellectual contribution to the work, and approved it for publication.
